# Diagnosis and treatment of bbstetrics disseminated intravascular coagulation in resource limited settings

**DOI:** 10.4314/ahs.v22i1.24

**Published:** 2022-03

**Authors:** Helen C Okoye, Theresa U Nwagha, Angela O Ugwu, Ifeanyi E Menuba, Augustine N Duru, Emmanuel O Ugwu, Feanyichukwu U Ezebialu, Stephen C Eze, Aloysuis O Ugwu

**Affiliations:** 1 Department of Haematology and Immunology, College of Medicine, University of Nigeria Ituku Ozalla campus, Enugu; 2 Department of Obstetrics and Gynaecology, College of Medicine, University of Nigeria Ituku Ozalla campus, Enugu; 3 Department of Obstetrics and Gynaecology, College of Medicine, Chukwuemeka Odumegwu Ojukwu University, Awka; 4 Department of Obstetrics and Gynaecology. Lagos University Teaching Hospital, Idi-araba

**Keywords:** Investigations, DIC, Obstetrician, diagnosis, treatment

## Abstract

**Background:**

Disseminated intravascular coagulation (DIC) is one of the commonest causes of abnormal bleeding during pregnancy and puerperium. Its successful management is a challenging feat in resource limited settings (RLS).

**Aim:**

To determine Obstetricians' approach in diagnosing and treating obstetrics DIC in a RLS

**Method:**

A semi-structured pre-tested 4-sectioned questionnaire was used to collect demographic data of Nigerian obstetricians and data on their practice in the diagnosis and treatment of obstetrics DIC.

**Results:**

A total of 171 obstetricians responded. Preeclampsia was the most frequent cause identified (70.2%) followed by postpartum haemorrahge (58.3%). Platelet count determination was the test mostly used (95.9%) to make a diagnosis of DIC whereas, antithrombin assay was the least (20.6%) requested investigation. While about two-third would monitor the evolution of DIC, a little less than half of the obstetricians would not repeat laboratory testing more than every 2 days, reason mainly (61.8%) due to patient's financial constraint. Almost three-quarter of them preferred fresh whole blood as the first line of treatment of DIC.

**Conclusion:**

DIC remains a challenge in the obstetrics practice in RLS especially in investigations, monitoring and index of suspicion for non-overt DIC.

## Introduction

Obstetric hemorrhage remains a leading cause of maternal morbidity and mortality worldwide, especially in resource limited settings.^1^,^2^ Lethal obstetric hemorrhages may be classified into two subgroups: a) Persistent bleeding following vaginal/cervical lacerations, uterine atony or from ruptured uterus.^3^ b) Hemorrhage that ensues without an obvious source of bleeding as seen in Disseminated Intravascular Coagulation (DIC).

Disseminated Intravascular Coagulation (DIC) is a pathological disruption of finely balanced process of hemostasis characterized by systemic activation of blood coagulation, resulting in fibrin production and deposition, widespread formation of microvascular thrombi in small blood vessels (thrombosis) and plasmin activation, leading to excessive bleeding and multiple organ dysfunction.^4^ It is also known as consumption coagulopathy and is regarded as a critical care clotting catastrophe.^5^

The incidence of DIC in pregnancy is said to be 0.03% to 0.34%. (1) The risk is higher with fetal demise; placental abruption; amniotic fluid embolism; pre-eclampsia, HELLP syndrome; acute fatty liver and septic abortion.^6^,^7^ These conditions lead to an increased elaboration of maternal thrombin, platelet activation, and abnormal fibrinolysis.^8^

The management of DIC requires a good knowledge of its pathophysiology, awareness of the risk factors and early recognition of the condition. A complement of trained personnel and access to modern therapies is also essential in the diagnosis and treatment of DIC. These components are scarce in resource limited settings and often lead to a delay in the diagnosis of obstetric DIC until a late stage, resulting in adverse maternal outcome including massive blood transfusion, hysterectomy, and maternal mortality.^7^

The basic step in the diagnosis and treatment of obstetric DIC is to identify patients at risk for this complication and to monitor them closely with both clinical and laboratory assessments.

Presently, there is no acceptable definition of laboratory abnormalities for DIC in pregnancy.^1^ Pregnancy is associated with an increase clotting and increase fibrinolytic capacity therefore laboratory findings in DIC need to be interpreted within the context of pregnancy specific values.^1^

Laboratory tests during DIC demonstrate progressively decreasing platelet count and fibrinogen, prolongation of the prothrombin time (PT) and activated partial thromboplastin time (APTT), increasing fibrinogen-degradation/fibrin-degradation products and D-dimer concentrations.^1^,^9^

The International Society on Thrombosis and Haemostasis (ISTH) and the Japanese Association of Acute Medicine (JAAM) have established diagnostic criteria for overt DIC. The scoring system comprised routinely available laboratory tests, including platelet count, prothrombin time, fibrin-related marker (D-dimer) or fibrinogen degradation products (FDPs) and fibrinogen. The JAAM diagnostic criteria categorically defined for sepsis induced DIC, included criteria for diagnosing of systemic inflammatory response syndrome (SIRS).^10^ These diagnostic criteria have high sensitivity (93%) and specificity (98%) as regards the diagnosis of DIC.^11^ The platelet count and prothrombin time (PT) tests could be used in absence of the other criteria to determine a patient with sepsis induced coagulopathy (SIC). A reduction in platelet and a prolongation of the PT in the presence of an underlying obstetric emergency should alert the managing physician of a possible DIC. 10

The D dimer has been used over the years to make a diagnosis of venous thromboembolism (VTE) and sepsis. It's a negative predictor of VTE. This means that levels of D- dimer within normal limits precludes a diagnosis of DVT while elevated levels of D- dimers connotes a possibility of VTE. However, D- dimers being a maker for fibrin formation and fibrin degradation is often raised in some conditions such as recent surgery, sepsis and inflammatory conditions.^12^,^13^ It has been shown that increasing D-dimer levels do not correlate with increasing severity from SIC rather a prolongation of the PT and a reduction in platelet count has been shown to lead to increasing mortality from SIC.^14^ This further buttresses the need for platelet count and PT especially in RLS where other laboratory investigations could not be obtained.

Of recent, there is a tilt towards the use of viscoelastic methods such as thromboelastography and thromboelastometry in the obstetric setting. 15,16 Its use has however not been systematically evaluated for use in obstetric DIC.

In the management of DIC, a multi- disciplinary approach involving the hematologists, obstetricians and neonatologists is necessary in order to optimize management and improve feto-maternal outcomes. The cornerstone of the management of DIC is the specific and vigorous treatment of the underlying disorder.^17^

In pregnancy-associated DIC, the principal aim is to address the obstetric abnormality. Once this is corrected, the DIC will usually subside. However, adjunctive supportive treatment specifically aimed at the coagulation abnormalities may be required in some cases particularly replacement of blood products.^6^

Prompt initiation of appropriate blood component replacement should be done using existing guidelines proposed in the management of coagulopathy associated with postpartum hemorrhage.^3^

Considering the dearth of manpower, modern facilities, blood and blood products in resource poor settings, this study is set out to determine the approach used by obstetricians working in such settings in the diagnosis and treatment of DIC.

## Materials and methods

This was a cross-sectional observational study conducted among Obstetricians in Nigeria over a period of 12 months between April 2018 and March 2019. Doctors working in the Obstetric units and those involved in the management of obstetrics cases both in public and private hospitals were eligible to participate in the study. The participants were recruited in their institutions as well as during the annual scientific gatherings of the obstetrics and gynaecology of Nigeria (SOGON). The study was done using a pre-validated, pre-tested structured questionnaire which was self-administered.

The questionnaire consisted of 3 major parts – 1^st^ part was data on socio-demography, the 2^nd^ was data on investigations for DIC, while the last part sought information on the treatment modalities.

Statistical analysis - Only completed questionnaires were analyzed. Information collected was transferred to an excel sheet and analysis was done using the Statistical Package for Social Sciences (SPSS) software version 21 (SPSS Inc., Chicago, IL). Statistical tools used include Chi square, and t tests for inferential statistics; confidence interval was set at 95% such that probability values less than 0.05 were considered as significant.

## Results

A total of 171 participants returned the questionnaires completely filled. We had 76.6% males and 23.4% females aged from 30 years and above. Most (67.2%) of the respondents had practiced for 5–15 years. Majority practiced in a tertiary facility (96.5%), public health facility (97.7%) and had seen 0–10 cases of DIC in the past year (81.2%), Details are as show in Table 1.

**Table 1:** Socio-demographic and other baseline variables of the study participants

Variable	Frequency (n)	Per Cent (%)
**Sex**		
Male	131	76.6
Female	40	23.4
**Age(Years)**		
30–39	63	40.1
40–49	66	42.0
≥ 50	28	17.8
**Designation**		
Consultant	82	48.0
Registrar	89	52.0
**Years of Practice**		
< 5	7	4.1
5–10	63	36.8
11–15	52	30.4
16–20	19	11.1
>20	30	17.5
**Place of Practice**		
Secondary	6	3.5
Tertiary	165	96.5
**Place of Practice**		
Public	167	97.7
Private	4	2.3
**Bed Capacity**		
<200	31	18.1
200–400	71	41.5
>400	69	40.4
**Delivery**		
<100	56	32.9
100–200	55	32.4
301–400	25	14.7
>400	34	20.0
**DIC cases**		
<10	138	81.2
10–50	11	6.5
>50	2	1.2
None	19	11.2

When inquired about the causes of obstetrics DIC, preeclampsia was the most frequent cause (70.2%) while retained products of conception and placenta praevia were the least common causes as depicted in Table 2.

**Table 2:** Common causes of obstetrics DIC as observed by the participants

**Variable Frequency (n) Percent(%)**
**DIC causes (Multiple Response n** **=324)**

In cases of suspected or overt DIC, platelet count, haemoglobin concentration (Hb) or packed cell volume (PCV), bedside clotting time and activated partial thromboplastin time (APTT) were the most frequently requested tests (Table 3), whereas, antithrombin and D-dimer were the least requested investigations.

**Table 3:** Investigations for suspected and evident DIC by the participants

**Variable Frequency (n) Percent(%)**
**Investigations (Multiple Response n=1** **454)**
Others (specify)

As shown in Table 4, 67.5% of respondents reported that monitoring the evolution of DIC was done by serial laboratory testing. Daily repetition of laboratory testing had the most prevalent frequency (43.0%), and the basic reason for not repeating laboratory test was financial constraints which accounted for 61.8%. Table 5 showed that most of our respondents (75.7%) did not look out for non-overt or chronic DIC.

**Table 4:** Monitoring of the evolution of DIC by the participants

Variable	Frequency (n)	Per cent (%)
**Monitoring evolution of DIC**		
Yes	114	67.5
No	55	32.5
**Repeat Lab Testing**		
Twice a day	12	10.5
Daily	49	43.0
Every other day	24	21.1
Weekly	8	7.0
Individualized protocol	21	18.4
**Reason for no Repeat Test**		
Financial constraint	34	61.8
Non-compliance from patients	2	3.6
Do not think it is needful	2	3.6
Inadequate lab service/Logistics	15	27.3
I do not know	2	3.6

**Table 5:** Investigation of potential DIC cases by the participants

Variable	Frequency (n)	Per cent (%)
**Look out for non- overt or chronic DIC in** **potential cases**		
Yes	41	24.3
No	128	75.7
**Test Used (Multiple Response n= 171)**		
ISTH, JAAM, JIMHW	10	27.0
Combination of tests	21	56.8
Non overt DIC cannot be diagnosed with current tests	5	2.9
Non overt DIC does not exist	1	0.6

ISTH International Society of Haemostasis and Thrombosis JAAM –Japanese Association for Acute Medicine JIMHW- Japanese Ministry of Health and Welfare

Information on the treatment option showed that most of our respondents used fresh whole blood (73.7%) as the first line treatment of choice while prothrombin complex concentrate was the least used (1.8%) as shown in Table 5.

In Table 6, platelet concentrate (25.7%) was the most frequent treatment option considered for 2^nd^ treatment while antifibrinolytics (4.7%) were used as 3^rd^ line treatment. Respondents reported that combined low Hb and platelet then preeclampsia (14%).^21^ This difference could be explained by the variation in incidence of obstetric complications between the low resource settings and the developed world.

**Table 6:** First line treatment options for DIC by the participants

Variable	Frequency (n)	Per cent (%)
**Treatment option for first line treatment**		
**Fresh whole blood**		
Yes	126	73.7
No	45	26.3
**Platelet concentrate**		
Yes	49	28.7
No	122	71.3
**Plasma/FFP**		
Yes	55	32.2
No	116	67.8
**Recombinant activated factor VII**		
Yes	7	4.1
No	164	95.9
**Prothrombin complex concentrate**		
Yes	3	1.8
No	168	98.2
**Fibrinogen**		
Yes	7	4.1
No	164	95.9
**Coagulation factors**		
Yes	9	5.3
No	162	94.7
**Antifibrinolytic agents**		
Yes	21	12.3
No	150	87.7

This study also shows tha t Obstetricians do not diagnose DIC appropriately although 95.9% do platelet count, 87.6% bed side clotting time, 82.4% APTT, Thrombin time, and PT/INR. Fibrinogen assay, D- dimer and Antithrombin are assayed for by 68.2%, 62.4%, 51.8%, 27.6%, and 20.6% of the participants respectively. These figures are low when contrasted with findings from higher income countries where most of the investigations are done routinely and serially.^21^ It is therefore pertinent from findings in this study to use the combination of platelet count and PT in RLS for diagnosis of obstetric DIC since other laboratory tests are not readily available.

The diagnosis of DIC is suspected clinically when bleeding occurs. Several investigations are often carried out to confirm the provisional diagnosis. Among the various tests done, the platelet count (95.9%) and haemoglobin estimation (90.6%) was the most requested investigations done by the participants. Bed side clotting time was done by 87.6% of respondents. The D- dimer test which serves as a negative predictor of thrombosis is not routinely done. It is obvious from this study that many laboratories in LRSs lack the facilities to screen for DIC coupled with the fact that patients often ‘pay out of pocket’ for medical care. These are the possble reasons why many of these tests were not routinely done in the study population.

About two thirds of the respondents monitored the evolution of DIC by serial blood tests. Repeat blood testing protocol varied with majority running the tests on a daily basis while less than a third of the respondents had an individualized protocol. Financial constraint was the major reason why repeat testing was not done. Sixty-one percent of respondents said that the choice of testing protocol was limited by financial constraints. Poverty affects about 70% of the Nigerian population.^22^ Making out of pocket expenditure for health care is rampant in Nigeria and further impoverishes the individual.^22^,^23^

Thus many investigations and treatment modalities are left undone because the patient could not afford them. The physicians are now left with using only clinical signs and symptoms to diagnose ailments including obstetric DIC. Most of the time, obstetricians look out for acute DIC only as buttressed by our study which showed that more than two thirds of the respondents do not screen patients for non-overt DIC.

The modified ISTH Scoring system is used to diagnose non-overt DIC. The parameters considered include: platelet count, PT, Fibrinogen, D dimer, antithrombin III, protein C and thrombin-antithrombin (TAT) complex.^24^ Also, the JAAM included the systemic inflammatory response syndrome (SIRS) criteria in their scoring system for DIC.^25^ Many of these tests are not readily available in most Nigerian laboratories and if available are done at prohibitive costs to the patient. In order to diagnose DIC in obstetric patients, the modified score which is sensitive and specific should be used to prevent obstetric deaths due to DIC.^26^

Furthermore, the first line treatment for obstetric DIC was found to be use of fresh whole blood (73.7%). This was followed by use of Fresh frozen plasma or fresh plasma (32.2%). The respondents have little knowledge about the use of purified factor concentrates (Prothrombin complex concentrate and Fibrinogen concentrate) and the recombinant activated Factor VIIa. These factor concentrates as well as component therapy are also not readily available in most of the centers.^27^ Thus the reason for use of fresh whole blood for the treatment of DIC in most hospitals. A different scenario is seen in higher in come clinical settings where patients with obstetric DIC have available resources and treatment modalities. This wide gap also infers that survival from DIC is better in such centers than in RLSs.

On the other hand, the use of fresh whole blood for the management of DIC is an excellent therapeutic modality in resource poor settings where other blood products such as FFP and Platelet concentrate or fibrinogen concentrate could not be procured. Fresh whole blood or whole blood is often administered to women with PPH because of their availability in resource poor settings.^28^ Women with postpartum haemorrhage develop DIC due to dilutional effect of the crystalloid and colloid infused to expand the plasma volume.^3^,^29^ The crystalloids or colloids do not contain the required coagulation factors and thus may worsen the bleeding problem. Coagulopathy from massive haemorrhage could also result from thrombocytopenia and loss of coagulation factors following bleeding.^30^

The respondents who had practiced for a longer period of time were more likely to monitor the evolution of overt DIC in their patients. This may have arisen from experience gathered over the years in their practice. Having a high index of suspicion of DIC in a pregnant woman is life saving.^31^ Women with obstetrics complications such as severe preeclapsia, abruptio placentae, septic abortion, amniotic fluid embolism and obstetric haemorrhage should be closely monitored for subsequent development of DIC.^31^ Clinical and laboratory evaluations to check for bleeding, shock or prolongation of the coagulation tests respectively should be dutifully employed.

The limitation of this country is that it is a single country study as all the participants were drawn from Nigeria. Thus, it may not be entirely representative of all the RLSs of the world. However, the collection of data from annual general meetings of SOGON where Nigerian obstetricians from both the public and private sectors are readily accessible makes the study sample representative of Nigeria obstetricians.

## Conclusion

The most important pregnancy related condition leading to bleeding with high mortality and morbidity rates is DIC, and pre-eclampsia is the leading cause in RLSs. In these settings, the diagnosis and treatment of obstetric DIC remains a big challenge as the obstetricians do not diagnose and treat DIC appropriately when compared to high income countries. This is because of lack of investigative tools as well as treatment options available to the obstetricians for the management of DIC.
